# Modeling of Safe Braking Distance Considering Pedestrian Psychology and Vehicle Characteristics and the Design of an Active Safety Warning System for Pedestrian Crossings

**DOI:** 10.3390/s25041100

**Published:** 2025-02-12

**Authors:** Yanfeng Jia, Shanning Cui, Xiufeng Chen, Dayi Qu

**Affiliations:** 1 School of Mechanical and Automotive Engineering, Qingdao University of Technology, Qingdao 266520, China; 901020220040@qut.edu.cn; 2 School of Civil Engineering, Qingdao University of Technology, Qingdao 266520, China; loser365@163.com (S.C.); chenhill7765@163.com (X.C.)

**Keywords:** pedestrian crossing, millimeter wave radar, video sensor, smart zebra crossing, safe braking distance modeling

## Abstract

Addressing the traffic safety issues caused by pedestrian–vehicle conflicts during street crossing, this study proposes optimization strategies from both theoretical and technical perspectives. A safety braking distance model is introduced, taking into account pedestrians’ psychological safety and vehicle braking processes. Additionally, an active safety warning system for crosswalks has been designed. This system features a modular design, including detection, control, alarm, and wireless communication modules. It can monitor, in real-time, the positions and speeds of pedestrians and vehicles, assess potential conflicts between them under various scenarios, and implement different warning strategies accordingly. Compared to mainstream variable message sign (VMS) warning systems, this proposed system shows significant advantages in terms of section-weighted total delay metrics. Through simulations involving 3000 pedestrian crossings and comparative analyses of vehicle speed, pedestrian speed, vehicle deceleration rate, and accident numbers before and after the application of the active safety warning system, it was found that the critical accident rate indicator decreased from 0.27% to 0.06%. The results demonstrate that the system effectively provides bidirectional warnings to pedestrians and vehicles, significantly enhancing the safety of pedestrian street crossings. This research offers new insights into addressing pedestrian crossing safety issues.

## 1. Introduction

As the pace of urbanization accelerates and traffic volumes continue to increase, the safety concerns of pedestrians, who are a vulnerable group in road traffic, have become increasingly important. Particularly at intersections or road sections without traffic signal control, risks to pedestrian safety when crossing the street are significantly high. Traditional measures such as signage, markings, and speed bumps have limited effectiveness in certain scenarios, especially under conditions of low illumination and reduced visibility (such as during nighttime or in foggy/rainy weather). Under these conditions, drivers may fail to detect pedestrians in a timely manner, thereby leading to increased incidences of traffic accidents. Urban road intersections serve as convergence points for vehicular and pedestrian traffic, making them hotspots for traffic accidents. Traffic intersection dangers continue to be a significant cause for worry in terms of safety. According to the data, these intersections are responsible for more than sixty percent of all urban accidents, the majority of which occur during signal transitions (ninety percent). It has been determined through analysis that pedestrians who begin crossing the street within the final five seconds of green signals are extremely unlikely to finish their journey before the light changes. As a result of this temporal mismatch, potentially hazardous situations arise in which people continue to remain in crosswalks while automobiles acquire the right-of-way into the intersection. Despite the fact that pedestrians make up the largest group of people who participate in traffic, they are also the most vulnerable to physical harm. Pedestrians account for twenty percent of all traffic casualties, with accidents involving intersecting streets accounting for more than half of these cases.

In order to address these concerns regarding safety, researchers from all around the world have devised a variety of protective devices. Early foundational work [1] introduced an active pedestrian protection system, which laid the groundwork for subsequent technological developments. A comprehensive crosswalk safety system was established by Jin et al. [2], incorporating illuminated signage, LED illumination, textual displays, and auditory alarms that activate when speeding vehicles approach pedestrians. Building on traffic signal optimization, Chowdhury et al. [3] developed a tracking-based dynamic-flash yellow-arrow strategy for permissive left-turn vehicles, improving pedestrian safety at intersections through real-time adjustments based on pedestrian movement patterns. An intelligent transport system, integrating carriage, sensor, traffic signal, and power components, has also been developed to enhance crossing safety [4]. Lu et al. [5] further improved pedestrian protection using movement-detection technologies, adaptive signal regulation, and physical barriers. However, the integration of IoT and sensor technologies in such systems presents challenges, as highlighted by Hasan et al. [6], who identified critical issues like sensor reliability, data privacy, and interoperability. Chen et al. [7] devised a bidirectional warning method using crosswalk illumination adjusted to vehicle speed and signal patterns, leveraging pedestrians’ rapid response capabilities. Complementing this, Soto et al. [8] proposed reducing unnecessary alerts in pedestrian protection systems through optimized vehicle-to-pedestrian (P2V) communication, enhancing reliability by minimizing false alarms. Yang et al. [9] developed a microcontroller-based safety device for dual-mode visual and audible alerts, while advancements in vehicle-infrastructure integration were surveyed by Hejazi et al. [10], outlining V2X-equipped smart intersection use cases and deployment challenges. Recent machine learning innovations include Zhou et al. [11], who used surveillance footage and Graph Convolutional Networks (GCN) to predict crossing intentions. Yazdani et al. [12] expanded AI-driven traffic control with an Intelligent Vehicle Pedestrian Light (IVPL) system, dynamically optimizing signal timing via deep reinforcement learning. Wang et al. [13] modeled pedestrian behavior at unmarked crossings using Maximum Entropy Deep Inverse Reinforcement Learning. Practical implementations of V2X technology were demonstrated by Hu et al. [14], who applied anti-collision warning systems at urban intersections. Behavioral studies have also informed system designs. Deb et al. [15] validated a pedestrian behavior questionnaire to assess crossing tendencies, while Mukherjee et al. [16] compared safety perceptions at signalized intersections. Wang et al. [17] advanced behavior prediction models by analyzing pedestrian–vehicle interactions, and Yuan et al. [18] incorporated psychological safety distances into risk assessment algorithms. Patel et al. [19] developed a proactive safety evaluation framework combining surrogate safety measures and non-compliance analysis, enabling quantitative risk assessments. Further advancements include an AI edge-computing wearable device with 90% accuracy for visually impaired users [20], a collision prediction FCW system [21], and, as Wang [22] designed, a VMS-based early warning system for unmarked crossings. Uchida et al. [23] enhanced the Pedestrian–vehicle Collision Avoidance Assistance System (P-VCASS), calculating danger levels for multiple pedestrians, which were validated through experiments.

In summary, existing models for safe stopping distances primarily consider the lead vehicle as a reference point and incorporate the driver’s reaction time into calculations, but they lack consideration of pedestrian psychological safety. Moreover, most scholars designing pedestrian crossing safety systems have not adequately addressed solutions for the signal transition period, and lane-specific fine control during warning periods remains underexplored. This research presents an enhanced approach to pedestrian crossing safety during signal transitions, integrating pedestrian psychological factors and vehicle braking dynamics through two key models: an improved stopping sight distance model and a pedestrian crossing psychological model. Building on these foundations, we develop a comprehensive vehicle safety braking distance model that synthesizes human behavioral patterns with vehicle performance characteristics, culminating in the design of a V2I (Vehicle-to-Infrastructure)-based active safety warning system for pedestrian crossings.

The paper’s structure encompasses five main sections: Section 2 details the refinement of existing parking safety distance models, and introduces our novel vehicle safety braking distance model, including comparative safety performance analysis. Section 3 outlines the warning system architecture, detailing its detection, control, alarm, and wireless communication components. Section 4 and Section 5 present the system simulation methodology and result analysis, respectively, while Section 6 provides conclusions and future research directions.

## 2. Safety Braking Distance Model Considering Pedestrian Crossing Psychology and Vehicle Characteristics

### 2.1. Pedestrian Waiting Time

The duration of pedestrian waiting time is key in the psychology of pedestrian crossings. Extended pedestrian waiting times heighten the likelihood of impatience and restlessness, hence increasing the probability of irresponsible roadway crossings. Reference [24] indicates that the greatest waiting time for pedestrians to cross the street is around 40 to 50 s. It was thought that a tolerable waiting time for most pedestrians in China is 50 s [25]. Combining relevant studies [26,27] and the characteristics of pedestrian waiting time, it can be observed that the relationship between pedestrian waiting time and the probability of reckless crossing is highly similar to the S-shaped curve model of population growth in biology. Therefore, the population growth model in biology can be modified to construct a probability influence coefficient, as shown in Equation (1), which can reflect the impact of pedestrian waiting time on the probability of reckless crossing.

(1) $$\zeta =\frac{k}{1+(\frac{k}{k_{0}}-1)\exp(-r_{k}t_{w})}+1$$

where ζ is the probability influence coefficient, and *k* is the psychological tolerance limit of pedestrians, which can take values between 0 and 2. Larger values indicate that pedestrians are closer to their psychological tolerance limit; *k*_0_ is the initial psychological perception of pedestrians; *r_k_* is the growth rate of psychological tolerance, which is related to pedestrian behavior; *t_w_* is the pedestrian waiting time.

**Figure 1:**
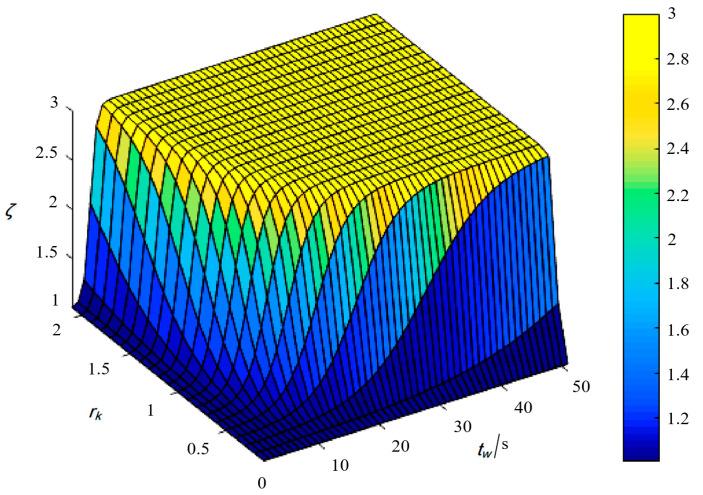
Three-dimensional surface plot of the probability influence coefficient ζ.

When the psychological tolerance limit *k* is 2, and the initial psychological perception *k*_0_ is 1, the function graph of the probability influence coefficient ζ, with respect to the growth rate of psychological tolerance *r* and pedestrian waiting time *t*, is shown in Figure 1. It can be observed that ζ increases with the increase in *r* and *t*, and the curvature also increases accordingly.

**Figure 2:**
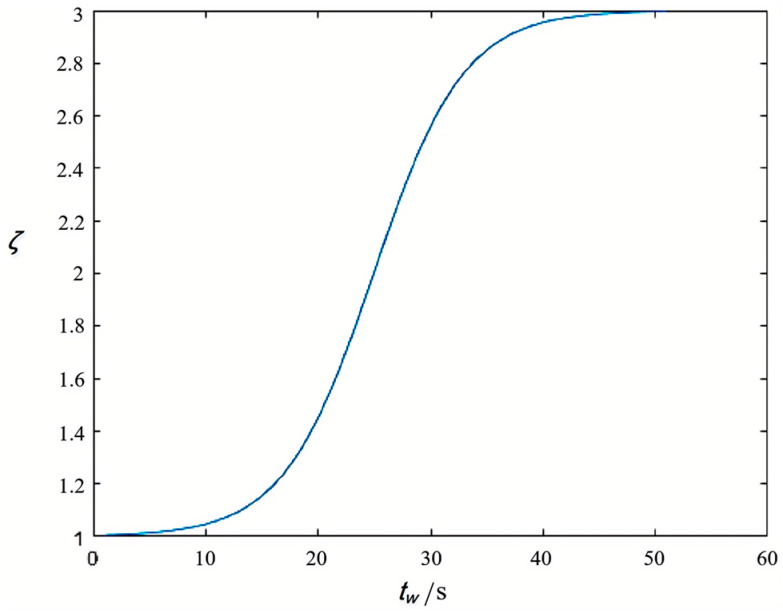
Image of the probability influence coefficient *ζ* function at *r_k_* = 25%.

Figure 2 depicts the probability influence coefficient as a function of pedestrian waiting time when *r_k_* is 25%. The graph illustrates three separate periods in the correlation between the likelihood of hazardous crossings and pedestrian waiting duration: the likelihood of hazardous crossing diminishes when pedestrian waiting time is under 20 s; between 20 and 35 s, the probability of risky crossing increases rapidly; and after 35 s, the probability of risky crossing approaches its maximum.

### 2.2. Safe Braking Distance Considering Pedestrian Psychology

In instances of conflict between a vehicle and a pedestrian, if the car brakes merely to meet the minimum safe braking distance, its excessive speed may result in stopping too near the pedestrian, which will have a considerable psychological impact on pedestrians. Consequently, when a vehicle and a pedestrian are in conflict, it is essential to guarantee that the vehicle can brake safely while simultaneously accounting for the pedestrian’s psychological safety distance. This is to prevent the vehicle from stopping too close to the pedestrian, which can cause fear and other unpredictable dangerous behaviors such as sudden movements or running away.

The psychological safety distance *L_p_* for pedestrian crossing is given by [28]:

(2) $$L_{p}=v_{i}(t_{r}+\frac{nL_{c}}{v_{p}})+C_{s}$$

The variables represent the following: vehicle speed in each lane (*v_i_*), total lane count (*n*), pedestrian response interval (*t_r_* = 1.8 s average), and vehicle-to-pedestrian clearance (*C_s_* = 3–5 m).

The pedestrian psychological safety model, initially constructed for constant-velocity scenarios, requires deceleration compensation. During braking, vehicle speed diminishes rapidly, rendering air resistance negligible. Braking deceleration (*a_j_*) can be derived from force equilibrium:

(3) $$a_{j}=\frac{g}{\delta }(\alpha +\beta \pm \sigma )$$

where *δ* is the coefficient of rotational mass conversion, typically taken as 1.1 to 1.4.

Reference [29] states that the necessity for psychological safety distance is contingent upon the vehicle’s type and size. The expression is as follows:

(4) $$r_{s}=\frac{20K}{1+\frac{a_{0}b_{0}}{a^{*}b^{*}}}\cdot \frac{a_{0}b_{0}}{a^{*}b^{*}}$$

where *r_s_* is the psychological safety distance for pedestrians in relation to various vehicles. *K* is the distinctive parameter of the pedestrian, associated with parameters including gender, age, and risk-taking behavior, and is generally greater than 0. *a*_0_ denotes the length of the target vehicle, whereas *b*_0_ represents its height. a^*^ and b^*^ represent the length and height of the reference vehicle, respectively.

The vehicle’s deceleration is represented as a homogeneous deceleration process. Equations (2) and (3), which incorporate time, velocity, and acceleration variables, are integrated into the kinematic equations for uniform deceleration. Furthermore, taking into account the impact of vehicle type on the pedestrian’s safety perception and braking distance, Equation (4) is incorporated into the kinematic equations. The constant term *C_s_* in Equation (2) can be disregarded due to the incorporation of various factors and the supplementary parameter for vehicle type. Therefore, the pedestrian’s perceived safety braking distance $L_{p}^{\prime }$ can be expressed as:

(5) $$L_{p}^{\prime }=v_{i}(t_{r}+\frac{nL_{c}}{v_{p}})-\frac{a_{j}}{2}(t_{r}+\frac{nL_{c}}{v_{p}}{)}^{2}+\frac{20K}{1+\frac{a_{0}b_{0}}{a^{*}b^{*}}}\cdot \frac{a_{0}b_{0}}{a^{*}b^{*}}$$

### 2.3. Improvement of the Minimum Safe Braking Distance Model for Vehicles

The safe braking distance of a vehicle denotes the distance covered from the moment the driver initiates braking (i.e., applies the brake) until the vehicle comes to a complete stop. This distance is one of the important indicators for evaluating vehicle safety. The safe braking distance model is shown in Equation (6) [30]:

(6) $$S_{0}=\frac{v_{0}\cdot t_{0}}{3.6}+\frac{v_{0}^{2}}{2g(\alpha +\beta )\cdot 3.6^{2}}$$

The safe braking distance (*S*_0_) calculation incorporates multiple parameters: driver braking reaction time (*t*_0_ = 2.5 s, accounting for delayed response), gravitational acceleration (*g* = 9.8 m/s^2^), wetness coefficient (α = 0.4 for wet conditions), and roughness coefficient (*β* = 0.03–0.05).

Current stopping sight distance models primarily focus on driver reaction time, neglecting several crucial phases: brake pedal free travel, brake shoe-to-drum contact duration, and the interval required to achieve maximum deceleration through complete pedal depression. Though brief, these latter components warrant inclusion in comprehensive modeling.

**Figure 3:**
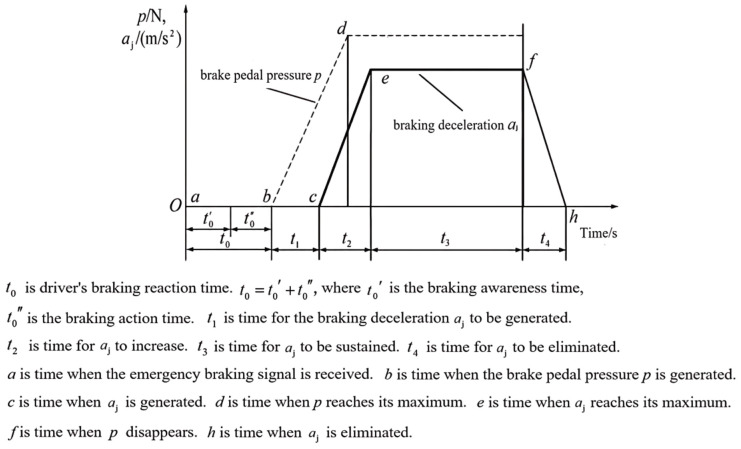
Vehicle braking process.

As illustrated in Figure 3, the complete vehicle braking sequence encompasses multiple stages. Emergency braking initiation involves hazard recognition ($t_{0}^{\prime }$) followed by response time ($t_{0}^{\prime \prime }$), with pedal engagement occurring at point *b* after point *a*. The total reaction interval *t*0 ($t_{0}^{\prime }\;$+${\;t}_{0}^{\prime \prime }$) spans from points *a* to *b*. Subsequently, time *t*_1_ elapses before deceleration onset at point *c*, compensating for mechanical gaps between brake components. Maximum deceleration achievement requires additional time, *t*_2_, reaching point *d*. The sequence concludes with the pedal release duration *t*_3_ (terminating at point *f*) and deceleration dissipation period *t*_4_ (concluding at point *h*).

Equation (7) introduces an enhanced stopping sight distance model that extends the foundation established in Equation (6). This refinement incorporates multiple mechanical and environmental factors: brake pedal travel dynamics, brake component clearance, deceleration build-up interval, and road gradient effects.

(7) $$S_{0}^{\prime }=\frac{v_{0}}{3.6}\;(t_{0}+t_{1}+\frac{t_{2}}{2})+\frac{v_{0}^{2}}{2g(\alpha +\beta \pm \sigma )\cdot 3.6^{2}}+l_{0}$$

where $S_{0}^{\prime }$ is the improved stopping sight distance; σ is the longitudinal slope of the road (positive for uphill and negative for downhill); and *l*_0_ is the safety distance, taken as 5 m. (*t*_1_ + *t*_2_) is generally taken as 0.2 to 0.9 s.

### 2.4. Modeling Safe Braking Distance Considering Human and Vehicle Characteristics

A comprehensive safety metric is produced as a result of the combination of the enhanced parking visibility model and the pedestrian psychological braking distance model. A weighting factor *ρ*, which must be more than or equal to 0.5, provides a balance between the priority of vehicles and pedestrians in various places, resulting in the human–vehicle characteristic safety braking distance *S*:

(8) $$S=\rho S_{0}^{\prime }+(1-\rho )L_{p}^{\prime }$$

To guarantee a vehicle can brake safely while considering the psychological effects on pedestrians, and to prevent the safe stopping distance from becoming unduly large and losing practical relevance, *ρ* is typically no less than 0.5. By disregarding constant terms and replacing *v_i_* in Equation (5) with the initial velocity *v*_0_ for each lane, we derive the model for safe braking distance that accounts for human and vehicle characteristics, which is:

(9) $$\begin{array}{c} S=\rho \left[\frac{v_{0}}{3.6}\;(t_{0}+t_{1}+\frac{t_{2}}{2})+\frac{v_{0}^{2}}{25.92(\alpha +\beta \pm \sigma )}\right]\;+\\ (1-\rho )\left[v_{0}(t_{r}+\frac{nL_{c}}{v_{p}})-\frac{g}{2\delta }(\alpha +\beta \pm \sigma )(t_{r}+\frac{nL_{c}}{v_{p}}{)}^{2}+\frac{20K}{1+\frac{a_{0}b_{0}}{a^{*}b^{*}}}\cdot \frac{a_{0}b_{0}}{a^{*}b^{*}}\right] \end{array}$$

**Figure 4:**
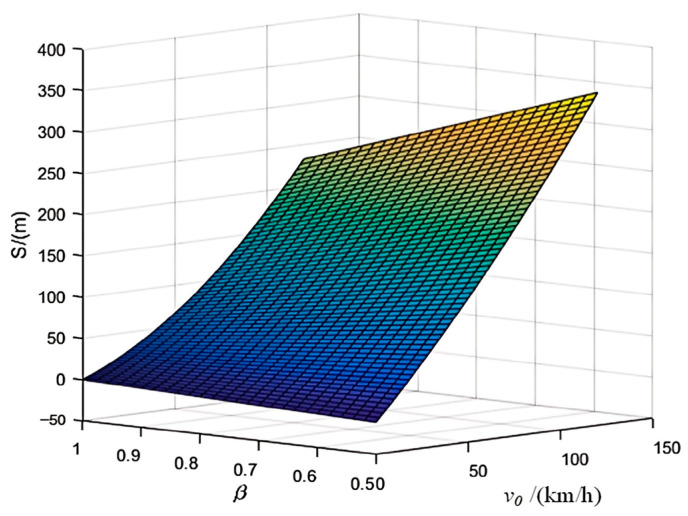
Surface of Vehicle Safety Braking Distance Considering Human–Vehicle Characteristics.

To intuitively assess the impact of the initial running speed *v*_0_ and the weight ρ of the vehicle on the model, other variables are held constant. This allows for the derivation of a vehicle safety braking distance surface that accounts for the characteristics of both pedestrians and vehicles, as illustrated in Figure 4.

### 2.5. Model Comparison and Analysis

Through the use of a comparative numerical analysis, our proposed human–vehicle integrated safety braking distance model is evaluated in comparison to three current frameworks. These frameworks include the parking visibility model, its upgraded form, and the pedestrian psychological braking distance model. To ensure that all models are comparable to one another, consistent parameters are used across the board. The only components that are subject to change are the initial vehicle velocity (*v*_0_) and weight factors.

Reaction time for the driver (2.5 s, delayed response scenario), rising time for the braking force (0.6 s), and gravitational acceleration (9.8 m per second squared) are all standard metrics. The following environmental parameters are specified: a wetness coefficient of 0.4, a roughness coefficient of 0.04, a flat road grade, and a standard safety margin of 5 m. A single-lane crossing scenario (*n* = 1, *L_c_* = 3.5 m) with a standard pedestrian velocity (*v_p_* = 1.5 m/s) is the primary focus of this investigation. Additional factors include the following: pedestrian reaction time (*t_r_* = 1.8 s), vehicle-pedestrian clearance (*C_s_* = 3 m), the rotational mass conversion coefficient (1.2, yielding *a_j_* = 3.6 m/s^2^), and the pedestrian characteristic value (*K* = 1). Both the target vehicle (4.8 m in length and 1.4 m in height) and the reference vehicle (five meters in length and 1.6 m in height) have dimensions that are equivalent to those of a typical compact car.

**Figure 5:**
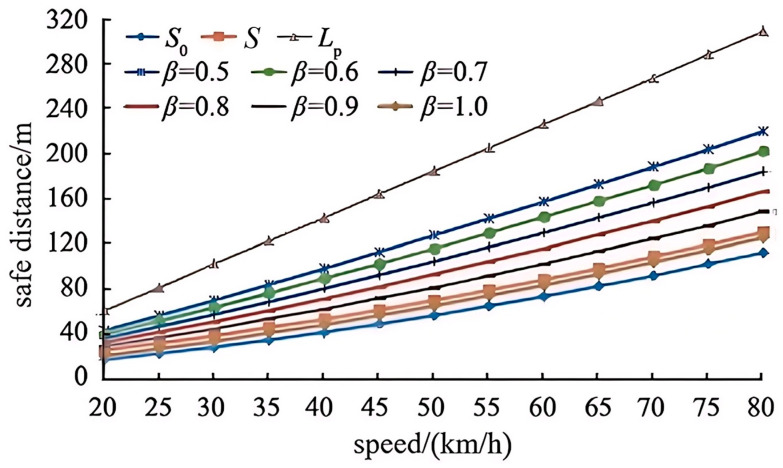
The safe distance solved for the vehicle safety braking distance model considering the characteristics of people and vehicles under different weights.

In the context of variable initial velocities (*v*_0_), safety distances are determined by the use of model computations. An additional analysis is performed on the human–vehicle integrated model, with weight factors ranging from 0.5 to 1.0, tested at 0.1 increments. The results of this analysis are displayed in Figure 5.

## 3. Design of Pedestrian Crossing Warning System

**Figure 6:**
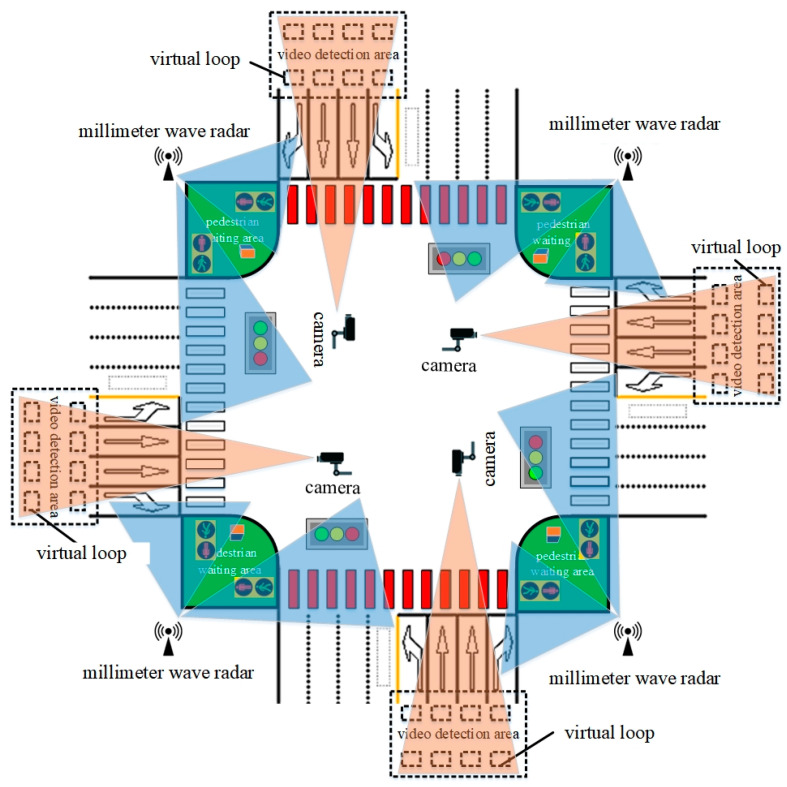
Composition of pedestrian crossing warning system.

A distributed architecture forms the foundation of the pedestrian safety alert system, integrating four key modules—detection, control, warning, and wireless communication components (illustrated in Figure 6). The implementation utilizes a hierarchical control structure: one primary controller coordinates with three subsidiary units positioned at crosswalk endpoints within pedestrian waiting zones, ensuring comprehensive coverage of intersection crossing areas. The system’s functionality encompasses pedestrian and vehicle monitoring, crossing duration computation, intra-system wireless data exchange, dynamic crosswalk illumination, and acoustic warning generation. The detection module is responsible for vehicle and pedestrian detection. Vehicle detection acquires speed information through virtual loops and video differential imaging techniques, while pedestrian detection is achieved through millimeter-wave radar ranging and height filtering for target identification. The control module, based on an MCU (Microcontroller Unit), receives real-time data from the detection module, analyzes pedestrian crossing times and vehicle speeds, dynamically calculates collision risks, and triggers warning strategies (such as voice prompts and intelligent crosswalk color switching). The warning module includes an intelligent LED crosswalk (ND16 series high-brightness indicators) and voice alert devices. The crosswalk color switches (red/yellow) based on vehicle speed, and the pedestrian waiting area posts provide voice prompts, enabling bidirectional warning. The wireless communication module utilizes IoT technology (UART interface) to synchronize data between modules (such as remaining signal time, vehicle/pedestrian positions), ensuring coordinated system operation.

**Figure 7:**
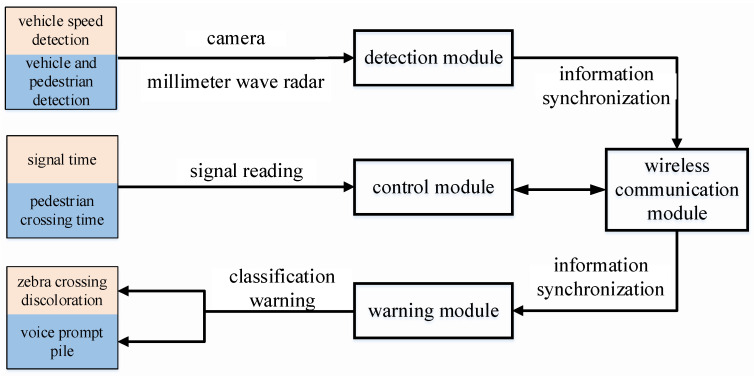
Schematic diagram of data flow interaction between modules.

The data flow between modules involves the detection module sending vehicle speeds and pedestrian positions to the control module. The control module assesses risks, synchronizes information via wireless communication, and instructs the warning module to activate appropriate alert strategies as shown in Figure 7.

### 3.1. Detection Module

The detection module comprises two components: vehicle detection and pedestrian detection. The technology mitigates risky circumstances resulting from knowledge imbalance between walkers and cars by simultaneously detecting both entities.

#### 3.1.1. Vehicle Detection

**Figure 8:**
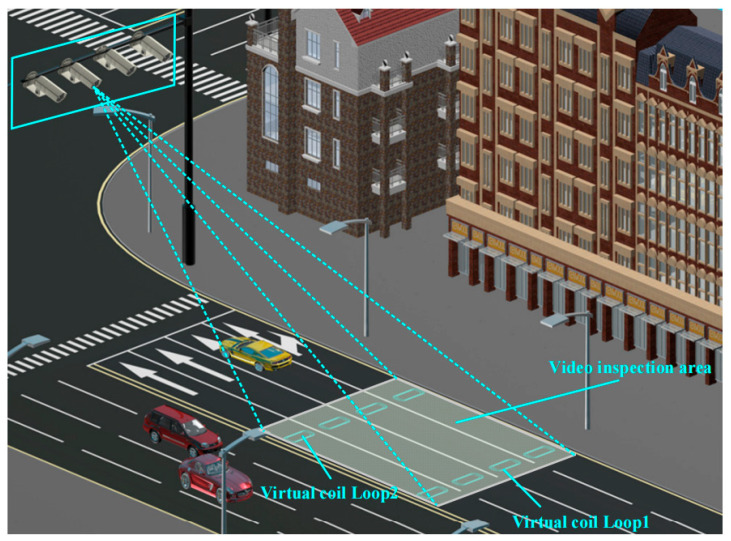
Vehicle detection part of the detection module.

Millimeter-wave radar detects objects by emitting electromagnetic waves and receiving the reflected waves from obstacles, capable of simultaneously detecting multi-lane and multi-target object information. It is used to determine whether vehicles are stopped before the stop lines at the entrance of each intersection. For the collection of vehicle speed and other information, a video detection method is employed, specifically utilizing the widely applied differential image method. A predefined video detection zone is set up in front of the intersection entrances, within which a pair of virtual loops (Loop 1, Loop 2) are designated for speed detection, replicating the speed measurement function of traditional dual-loop detectors. Through differential image analysis of the recorded video material, the mean velocity of cars traversing the two virtual loops (Loop 1, Loop 2) may be determined. Figure 8 depicts the vehicle detecting part.

By extracting the features of two consecutive frames, that is, by tallying the RGB (Red, Green, Blue) values of these two adjacent frames, and then performing a differential analysis on the two sets of RGB statistical values, the signal generated as vehicles pass through Loop 1 and Loop 2 can be obtained. The differential, *d*_RGB_, for the image RGB statistical values is denoted as:

(10) $$d_{\mathrm{RGB}}=\sum_{x=0}^{M-1}\sum_{y=0}^{N-1}\mathrm{RGB}\left[f(x,y,t)\right]-\sum_{x=0}^{M-1}\sum_{y=0}^{N-1}\mathrm{RGB}\left[f(x,y,t-1)\right]$$

where *f* (*x*, *y*, *t*) denotes the pixel value at the coordinates (*x*, *y*) in the *t*-th frame of the image. RGB(•) is the statistical function representing the red, green, blue, and grayscale values at the coordinates (*x*, *y*). *M* represents the length of the image, whereas *N* denotes its width.

**Figure 9:**
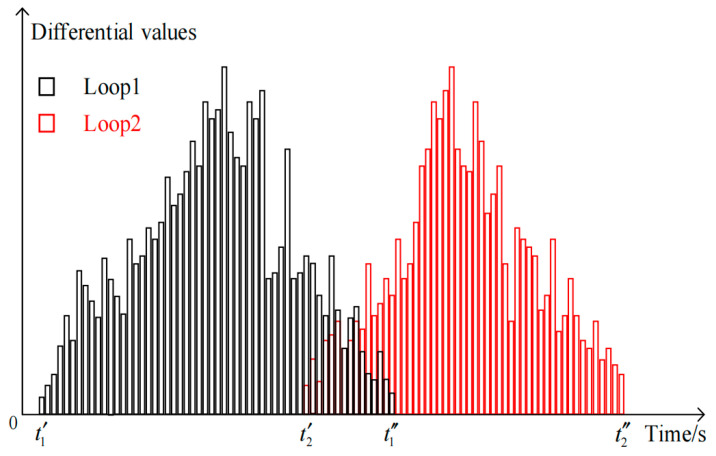
Image differential signal output generated when the vehicle passes through virtual Loop 1 and Loop 2.

The differential signal produced by the image when a vehicle traverses Loop 1 and Loop 2 encapsulates the complete sequence of the vehicle’s passage through the virtual loop, as illustrated in Figure 9.

The average speed *v_a_* of a vehicle traversing Loop 1 and Loop 2 is determined using the subsequent formula:

(11) $$v_{a}=\frac{1}{2}\left(\frac{d}{t_{2}^{\prime }-t_{1}^{\prime }}+\frac{d}{t_{2}^{\prime \prime }-t_{1}^{\prime \prime }}\right)$$

where *d* represents the distance between Loop 1 and Loop 2. $t_{1}^{\prime }$ and $t_{1}^{\prime \prime }$ are the times when the vehicle arrives at and departs from Loop 1, respectively. $t_{2}^{\prime }$ and $t_{2}^{\prime \prime }$ are the times when the vehicle arrives at and departs from Loop 2, respectively.

Based on the actual traffic conditions at each intersection, the position of the virtual loop in the video detection zone is determined. According to practical engineering cases, the virtual loop is generally set about 40 m in front of the stop line. Upon establishing the location of the virtual loop, it is essential to compute the beginning operating speed, *v*_0_, at which the vehicle can safely halt from the virtual loop to the stop line. The vehicle’s safe speed can be assessed by comparing *v*_0_ with the average speed, va, of the vehicle traversing the virtual loop.

#### 3.1.2. Pedestrian Detection

**Figure 10:**
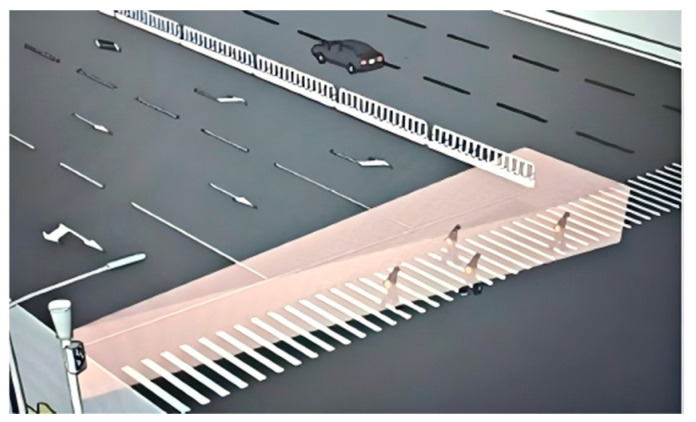
Pedestrian detection part of the detection module.

Pedestrian detection entails identifying and monitoring several targets throughout a designated region. A frequency-modulated continuous wave (FMCW) millimeter-wave radar, characterized by great sensitivity and considerable speed and distance measurement precision, is employed to guarantee data accuracy and reliability. This radar possesses robust anti-interference capabilities and can persistently monitor and measure the distance and velocity of many targets. Figure 10 illustrates the pedestrian detecting component.

Given the heightened sensitivity of radar detection, it is imperative to exclude other entities resembling pedestrians. To ascertain if a target is a pedestrian, it denotes the distances from the radar to the upper and lower extremities of the target as *l*_1_ and *l*_2_, respectively, and the angles from the radar to these extremities as *θ*_1_ and *θ*_2_. The height h of the identified target can be computed as:

(12) $$h=\sqrt{{l_{1}}^{2}+{l_{2}}^{2}-2l_{1}l_{2}\cos(\theta_{1}+\theta_{1})}$$

Given that the typical height of pedestrians ranges from 0.7 to 2 m [18], *h* can be assessed against this height range to identify and exclude targets that fall outside of it, specifically non-pedestrian targets. The positions of pedestrians can be ascertained by calculating the relative distance between them and the radar. The distance measurement can be articulated as follows, when there is relative motion between pedestrians and radar:

(13) $$r=\frac{Tc}{8\Delta F}\left(\Delta f^{-}+\Delta f^{+}\right)$$

where *r* is the relative distance between the radar and the pedestrian. *T* is the signal period. *c* is the speed of light. $\Delta f^{-}$ and $\Delta f^{+}$ are the differential signal frequencies of the negative and positive frequency segments after signal overlap, respectively. Δ*F* is the frequency modulation bandwidth.

The pedestrian’s speed relative to the radar can be estimated as follows [31]:

(14) $$\Delta f^{-}=\Delta f+\frac{2f_{0}v_{b}}{c}$$

(15) $$\Delta f^{+}=\Delta f-\frac{2f_{0}v_{b}}{c}$$

(16) $$v_{P}=\frac{c}{4f_{0}}\left(\Delta f^{-}-\Delta f^{+}\right)$$

where Δf is the difference in frequency between the sent and reflected signals. *f*_0_ is the transmitted signal’s center frequency. *v_b_* is the target’s speed relative to the radar. *v_p_* represents the pedestrian’s speed.

Detecting pedestrian position and speed allows for a precise understanding of pedestrians’ circumstances in each lane.

This information, combined with the vehicle speeds detected in each lane, allows for targeted, lane-specific detailed warning and control measures.

### 3.2. Control Module

The control system architecture incorporates six functional components centered around an MCU (micro-controller unit): the pedestrian signal timing monitor, vehicle velocity analysis, pedestrian movement detection, crosswalk illumination management, audio alert control, and the acquisition of the remaining time. System activation triggers at the 10 s mark of the remaining green signal phase. Upon detecting crossing intent, the system evaluates completion feasibility within the available time window. When completion appears unfeasible, an audio deterrent activates. Should pedestrians proceed despite warnings, the adaptive crosswalk system engages, implementing situation-specific color schemes to alert both pedestrians and approaching vehicles.

The pedestrian crossing duration (*t_p_*) calculation determines safe passage possibility, utilizing the following formula:

(17) $$t_{p}=\frac{L_{c}k}{v_{p}}+\frac{x^{\prime }+y^{\prime }}{s}$$

where *L_c_* is the lane width, is the number of lanes, and are the numbers of pedestrians on the left and right sides of the crosswalk, respectively. *S* is the safety coefficient, which is commonly set to 5.

**Figure 11:**
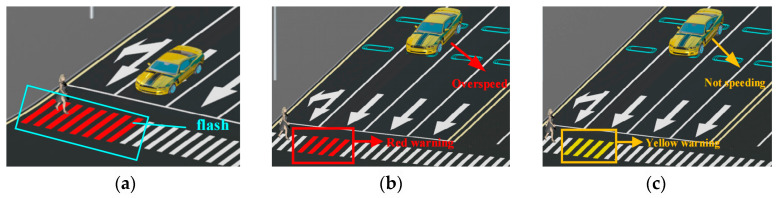
Three situations of intelligent zebra crossing warning. (**a**) Scenario 1: zebra crossing red light flashing. (**b**) Scenario 2: zebra crossing red warning. (**c**) Scenario 3: zebra crossing yellow warning.

By comparing the pedestrian’s crossing time *t_p_* to the remaining green light duration u, the system can assess whether the walker can finish the crossing or reach the central safety island. When the voice warning is ineffective and the pedestrian has already entered the crosswalk, the intelligent crosswalk warning can be classified into three scenarios based on the state of the *k* vehicles in the next lane, as seen in Figure 11.

Scenario 1: When a vehicle is halted, the control module will activate the pedestrian crossing signal to flash red for both the lane occupied by the pedestrian and the next lane ahead (see to Figure 11a). This inhibits the vehicle operator from commencing movement when the signal alters owing to inattention or insufficient observation, potentially resulting in a traffic collision.

Scenario 2: If no cars are halted and a vehicle over the safe speed limit is approaching, the control module will activate the pedestrian crossing signal to flash red for the lane directly ahead (refer to Figure 11b). This alerts both the car and the pedestrian to the imminent peril.

**Figure 12:**
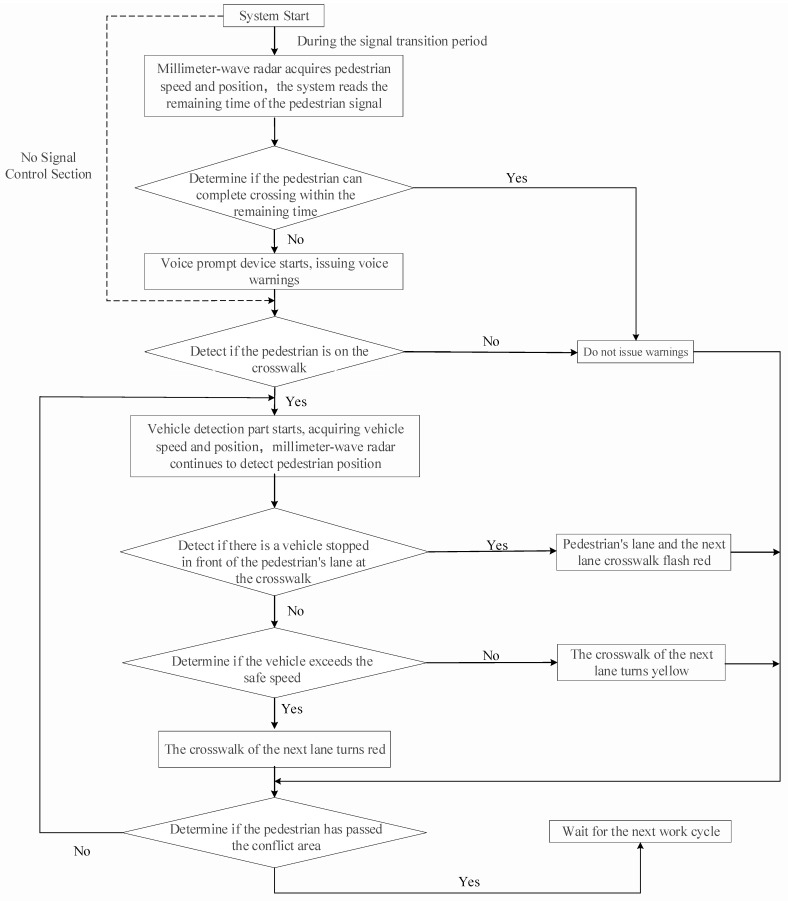
Control process of control module in warning system.

Scenario 3: In the absence of stopped vehicles, if a vehicle is approaching at or below the safe speed limit, the control module will activate the pedestrian crossing signal to flash yellow for the next lane (see to Figure 11c). This alerts the driver and the pedestrian to advance with prudence when it is safe to proceed. Figure 12 illustrates the control flow of the control module governing the complete pedestrian crossing operation.

### 3.3. Warning Module

**Figure 13:**
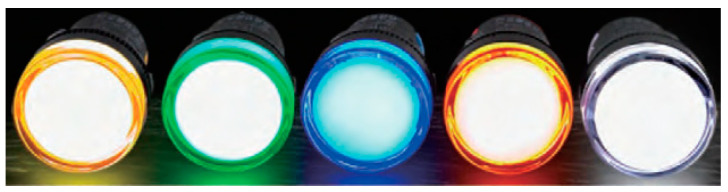
ND16 series LED highlight indicator effect.

Multiple feedback methods are incorporated into the warning system. These mechanisms include auditory alerts and an adaptive crosswalk display. Through the modification of control programs, audio functionality can make use of pre-existing intersection announcement systems. Alternatively, new audio devices can be deployed as required. Our selection of LED components for the intelligent crosswalk system, which substitutes traditional crosswalk markings, was informed by the findings of research that was published in [32]. These findings suggest that drivers are more sensitive to LED-embedded signage, particularly in low-speed pedestrian crossing zones. In order to fulfill the operating requirements of luminosity, visibility, durability, and energy efficiency, the LED high-brightness indicators from the ND16 series were chosen (Figure 13).

Following GB 5768.3-2009 [33] road marking standards, zebra crossings must maintain specific dimensional requirements, with lengths spanning 3–5 m, featuring alternating stripes of 45–60 cm width and 60 cm spacing. Implementing the minimum dimensional specifications, we establish an optimal crossing length of 300 cm with 45 cm stripe widths. The selected ND16 series LED high-brightness indicators, at 3 cm in diameter, are arranged in a precise five-row configuration within each stripe, maintaining 5 cm intervals between units. This geometric arrangement necessitates approximately 37 LED indicators per row (calculated as 300/(3 + 5)), culminating in a total of 185 LED high-brightness indicators per stripe (37 × 5 configuration).

**Figure 14:**
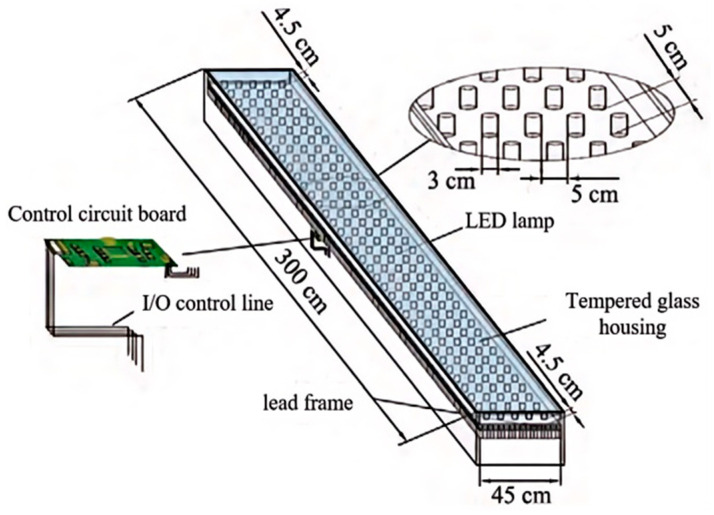
Intelligent zebra crossing structure in early warning module.

To ensure operational longevity and safety in real-world conditions, the intelligent crosswalk incorporates multiple protective elements through a sophisticated layered design. High-strength tempered glass encapsulation serves as the primary protective barrier, delivering optimal durability while maintaining essential transparency for LED visibility. The surface undergoes dual treatment processes: an anti-slip treatment to ensure secure footing for pedestrians and vehicle traction, complemented by an advanced anti-glare (AG) coating. This specialized AG treatment involves the application of proprietary chemical agents to the tempered glass surface, creating a matte diffusive layer that effectively mitigates LED-induced glare, enhancing visibility for both pedestrians and vehicle operators. The complete structural configuration and these integrated safety features are detailed in Figure 14.

### 3.4. Wireless Communication Module

**Figure 15:**
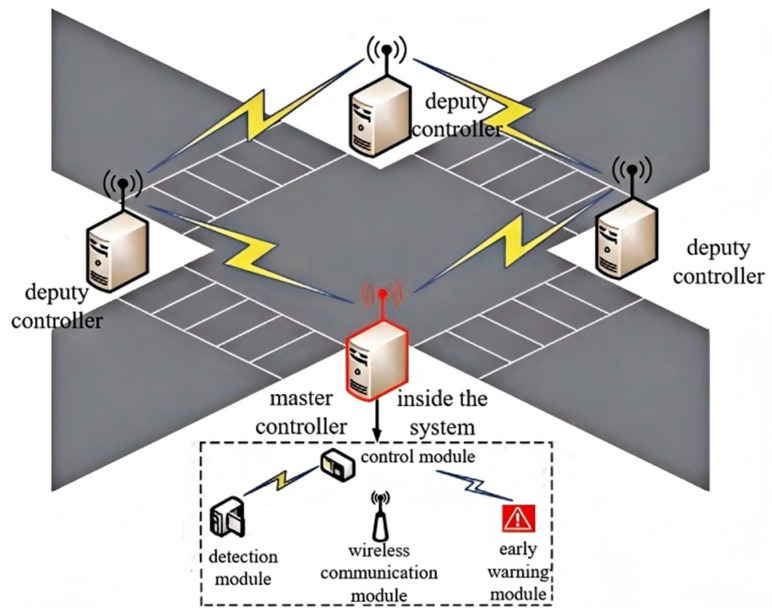
Wireless communication module in the system.

Wireless communication is chosen for data exchange within the system, as well as among various systems within the regional range, as shown in Figure 15. This is due to the fact that the installation of wired devices necessitates the destruction of roads. In addition, this choice is made in order to reduce the amount of construction and to increase the level of system intelligence.

Wireless communication predominantly transpires via the Internet of Things (IoT) module. The IoT module is incorporated into the system and interfaces with the microcontroller through a Universal Asynchronous Receiver–Transmitter (UART) connection. The IoT module enables information sharing across distributed systems at junctions and synchronizes data, including vehicle, pedestrian, and signal information, among various modules within the system, assuring coordinated operation of the entire system. The data transmitted inside the system encompasses residual signal duration, vehicular velocity, pedestrian crossing intentions, and the location and speed of people on the crosswalk.

## 4. Simulation Design of Early Warning System

### 4.1. Simulation Ideas and Assumptions

In order to verify the effectiveness of the designed pedestrian crossing active safety early warning system, a simulation comparison test before and after the application of pedestrian crossing active safety early warning system was designed.

1. Before application.

When there are vehicles in the lane ahead and pedestrians on the crosswalk, most pedestrians and vehicles will choose to mutually decelerate to avoid each other, although there are a few cases where they do not mutually yield.

2. After application.

- If the vehicle speed is less than or equal to 30 km/h, the vast majority of vehicles will decelerate and stop before the crosswalk. At this time, the crosswalk in front of the lane should be yellow. When pedestrians see the yellow crosswalk, they should prioritize crossing the lane before the conflicting vehicle, with only a very small number of pedestrians colliding with the vehicle.
- If the vehicle speed is greater than 30 km/h, the vehicle cannot safely stop before the crosswalk under any circumstances. In this case, most vehicles will decelerate and stop (but not before the crosswalk; they will stop on the crosswalk), with a small number of vehicles decelerating through. At this time, the crosswalk in front of the lane should be red. When pedestrians see the red crosswalk, the vast majority should stop and wait, with only a very small number continuing to walk forward and colliding with the vehicle.

In the simulation, both pedestrians and vehicles are assumed to be absolutely rational individuals who fully comply with the behavior of the pedestrian and vehicle. The simulation scenario is set as a pedestrian crossing channel on a road segment. Finally, after multiple rounds of simulation, the system outputs the speed, acceleration, and accident rate of pedestrians and drivers before and after the application of the pedestrian crossing active safety warning system for comprehensive comparative analysis.

### 4.2. Construction of the Simulation Environment

#### 4.2.1. Simulation Tools and Configurations

The simulation site was selected at a pedestrian crossing channel with moderate pedestrian traffic and good visibility, characterized by three lanes for motor vehicles and one lane for non-motorized vehicles. When constructing the simulation environment, the model building and parameter calibration were based on this location as a prototype. The virtual simulation was achieved through the combined use of 3Ds Max (2.24.2) and Unity 3D (2021.3) software. For the implementation of virtual simulations, the configuration of the PC includes a central processing unit (CPU)—the Intel i9-12900H (Intel Corporation, Santa Clara, CA, USA), featuring 14 cores and 20 threads with a base frequency of 5.0 GHz— and an NVIDIA RTX4060 (NVIDIA Corporation, Santa Clara, CA, USA) high-performance dedicated graphics card. This PC configuration satisfies the requirements for automatic storage and model rendering locally, eliminating the need to upload models and scenes to cloud servers for rendering and analysis once they have been constructed.

Given that the simulation scenario requires a limited number of models and a moderate level of detail for rendering, external standalone modeling using 3Ds Max was adopted. The SpeedRoad plugin within 3Ds Max enables one-click generation of road models. After downloading and installing the SpeedRoad plugin, a straight line (with the length corresponding to the required road length) is drawn in the 3Ds Max scene. By sequentially selecting MAXScript/Run Script from the menu bar and locating SpeedRoad, users can set parameters such as the number of lanes and width within the script, after which the road model is generated directly.

Once the road model configuration is completed, it is exported in the .FBX format. Following the setup of model properties, the model can then be imported into Unity 3D for further use. For the human and vehicle models within the traffic scene, existing model asset packages from Unity 3D can be directly utilized. After setting the basic parameters and initial positions of these models, their movements can be controlled via scripts.

#### 4.2.2. Implementation of Pedestrian Crossing Active Safety Warning System Function

**Figure 16:**
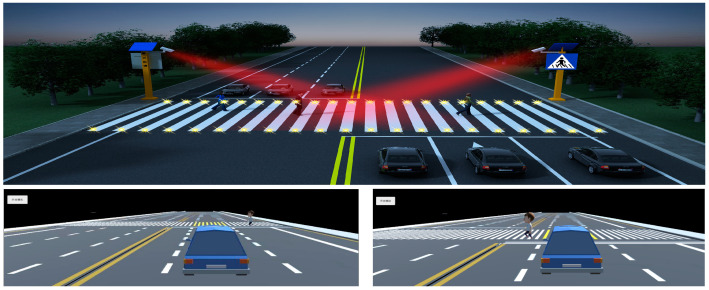
Schematic diagram of pedestrian crossing simulation without signal lights.

In the simulation scenario, multiple cubes (CUBE) are created and transformed into rectangular prisms by adjusting their parameters to match the dimensions of the zebra crossing on the road. These prisms are positioned over the zebra crossing locations in the road scene. The color changes in the zebra crossing are controlled based on the reading of vehicle movement speeds within the scene. The process is as follows: a simulation loop is established, which starts when the number of clicks to initiate the simulation is greater than or equal to zero. Initially, the zebra crossing appears white. If the vehicle speed exceeds 30 km/h, the zebra crossing turns red, indicating a failed safety status. Conversely, if the vehicle speed is less than or equal to 30 km/h, the zebra crossing displays yellow, signifying a successful safety status, the schematic diagram of the simulation scenario is shown in Figure 16.

**Figure 17:**
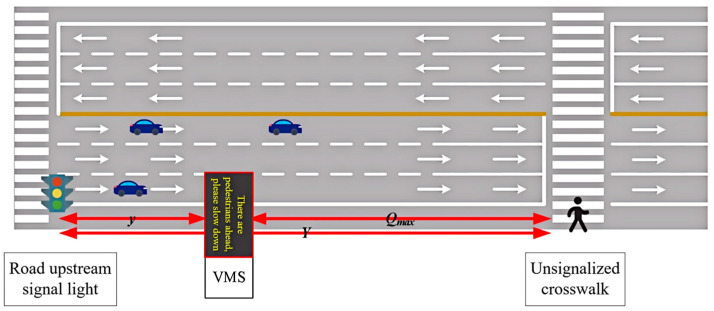
Schematic diagram of pedestrian crossing with VMS warning system.

In order to test the effectiveness of the early warning system, we choose to compare it with the current mainstream security early warning system based on variable message signs (VMS) in [34]. The schematic diagram of VMS early warning system is shown in Figure 17. Here, we use the same parameter settings and test environment to ensure the fairness and comparability among the models. Considering real-world scenarios where vehicles do not exceed moderate speeds while passing through areas with zebra crossings, the vehicle speed is set between 15 and 50 km/h. Based on existing research regarding pedestrian movement speeds, pedestrians’ movement speeds are set between 1 and 1.8 m/s. Given that the vehicle speed detection point is located 40 m before the stop line, in conjunction with safe speed and stopping sight distance models, the maximum deceleration for vehicles is set at 2.5 m/s^2^. Considering the maximum pedestrian movement speed and lane width, the maximum deceleration for pedestrians is determined to be 0.51 m/s^2^. For the sake of experimentation, it is assumed that both pedestrians and vehicles undergo uniform deceleration when slowing down.

## 5. Result Analysis and Discussion

Virtual simulations were conducted 3000 times in each of the two environments. After the simulations, the data were automatically recorded and analyzed to assess vehicle speed, pedestrian speed, vehicle deceleration, and the number of accidents.

1. Vehicle Speed Analysis

**Figure 18:**
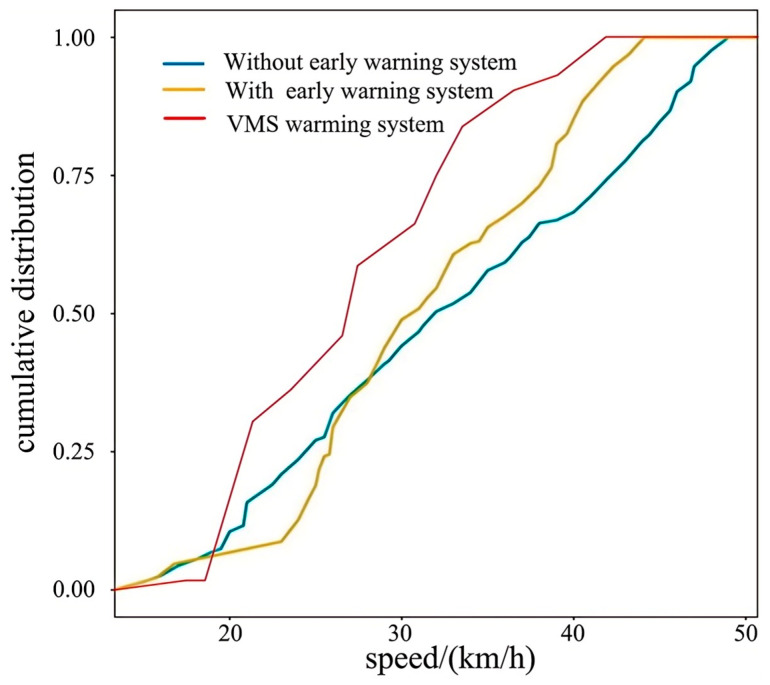
Cumulative vehicle speed distribution comparison chart.

The curve change in Figure 18 shows that the early warning system significantly reduces the speed concentration range. The slope of the curve without the early warning system is relatively flat, indicating that the speed distribution is relatively scattered and the driver’s speed selection is quite different. For example, the low cumulative ratio at 30 km/h indicates that most vehicles exceed this value. The curve with early warning system moves to the left as a whole and is steeper, and the cumulative proportion is higher at the same speed. For example, at 30 km/h, the cumulative proportion with early warning system is 0.5, while that without the early warning system is only 0.35, the difference is significant. This indicates that the early warning system encourages more drivers to reduce their speed actively, and the speed is concentrated in a lower range (such as 20–40 km/h). The vehicle speed concentration section under the VMS early warning system is the lowest, mainly around 20 km/h and 30 km/h, indicating that its effect may be affected by the efficiency of real-time information transmission or driver response habits. In the low-speed section of 25–30 km/h, the cumulative proportion of the two early warning system curves is higher than that of the non-early warning system, indicating that the early warning system can effectively increase the proportion of low-speed vehicles and reduce the risks associated with higher speed. This tendency towards safe speeds is likely because, in the pedestrian crossing environment without an early warning system, drivers mainly rely on their own experience to judge the road conditions, resulting in more scattered speed distributions, leading to a significant difference between low speed and high speed. When the pedestrian crossing active safety warning system is applied, the driver can use the color change in the intelligent zebra crossing system and VMS to assist in judging the traffic conditions, so that the speed distribution in the medium speed section is more concentrated. This also helps to ensure traffic safety, resulting in a slight reduction in the maximum speed, with the average speed slightly lower than the safe speed of 30 km/h.

2. Analysis of Vehicle Deceleration

**Figure 19:**
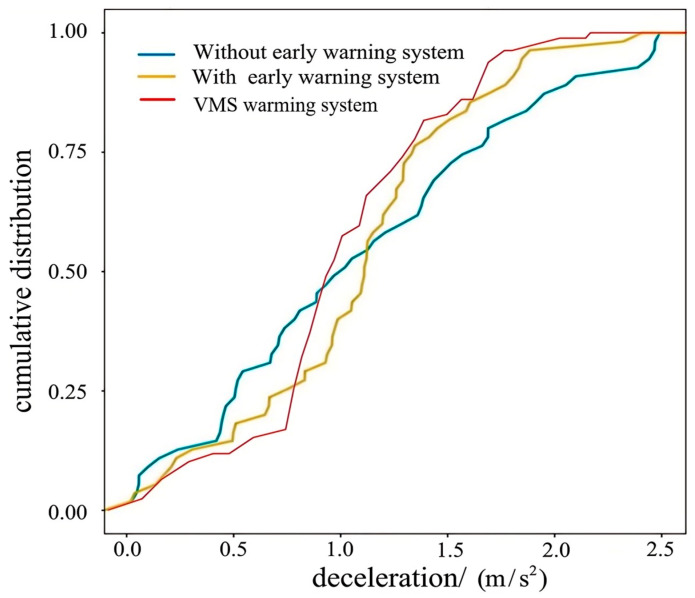
Comparison of the cumulative distribution of vehicle deceleration.

The cumulative distribution data of vehicle deceleration in Figure 19 indicate that, in the lower deceleration rate range (e.g., 0.0~0.5 m/s^2^), the changes in the three curves are relatively close, indicating that under the condition of low deceleration rates, the differences between different early warning systems are small. With the increase in deceleration rates, a difference between the curves gradually appears: in the range of 0.5 to 1.0 m/s^2^, the yellow curve and red curve begin to grow significantly higher than the blue curve, indicating that the two early warning systems can significantly reduce the deceleration rate of vehicles in this range. In the range of 1.0 to 2.0 m/s^2^, the yellow curve and red curve continue to show a better trend than the blue curve, but the red curve is more prominent at some points. In the range of 2.0 to 2.5 m/s^2^, the three curves tend to converge, indicating that the difference between different early warning systems decreases under the condition of high deceleration rates. In general, the early warning system and the VMS early warning system show significant advantages in reducing vehicle deceleration rates, especially in the range of medium deceleration rates (0.5 to 2.0 m/s^2^). Without the early warning system, the deceleration rate of vehicles is more widely distributed, and in the range of higher deceleration rates, its cumulative distribution is lower than the other two warning systems.

3. Analysis of Pedestrian Speed

**Figure 20:**
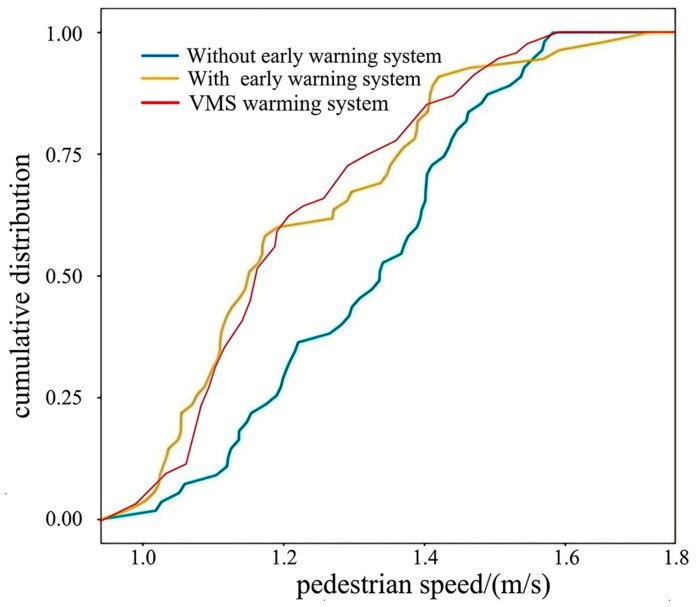
Comparison of the cumulative distribution of pedestrian speed.

The cumulative distribution data for pedestrian speeds in Figure 20 indicate that in the conventional pedestrian crossing simulation environment, the average pedestrian speed was 1.32 m/s, with a maximum speed of 1.58 m/s and a minimum speed of 1.02 m/s. This range of speeds is relatively large, possibly because pedestrians primarily rely on their own experience to judge road conditions, leading to both slow and fast crossing behaviors. The distribution of pedestrian speeds was relatively dispersed. In the pedestrian crossing active safety warning system simulation environment, the average pedestrian speed was 1.22 m/s, with a maximum speed of 1.72 m/s and a minimum speed of 0.98 m/s. Pedestrians’ performance in VMS warning system model is similar to their performance in the active safety early warning system model, with the speed distribution being more centralized towards moderate-to-slightly-lower speeds, and with fewer instances of rapid crossing. This indicates that when the pedestrian crossing active safety warning system is applied, pedestrians are able to use the color changes of the intelligent zebra crossing or VMS to assist in judging traffic conditions. Upon observing the color changes as a warning, most pedestrians tend to cross more cautiously, resulting in a more centralized distribution of pedestrian speeds towards moderate to low speeds. However, there are still a few pedestrians who, upon receiving the warning, choose to cross quickly, leading to a small number of pedestrians having speeds faster than usual.

4. Analysis of Weighted Total Delay and Traffic Accidents

**Figure 21:**
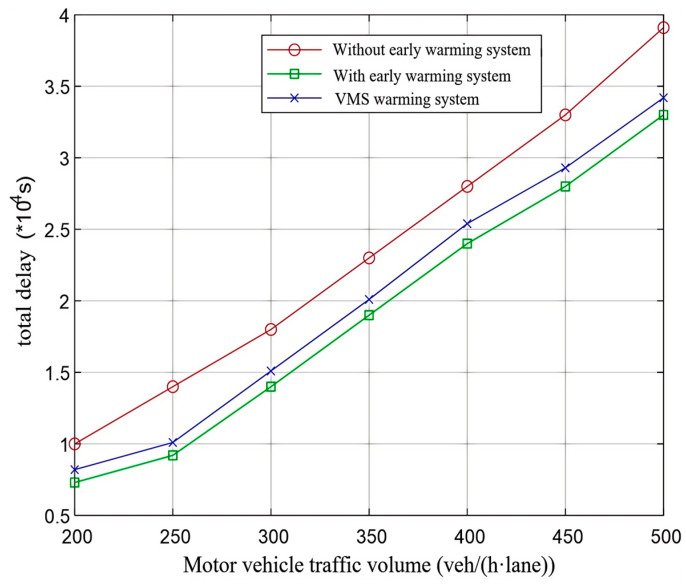
Comparison of weighted total delay of road section under different methods.

When calculating delays under yielding conditions at unsignalized crosswalks and assessing the effectiveness of optimizing pedestrian crossing regulations on such sections, it is crucial to account for the delays experienced by vehicles, pedestrians, and non-motorized vehicles concurrently. By applying the methodology from reference [35], these delays are assigned weights according to their significance to ascertain the section’s aggregate weighted delay. The outcomes for the aggregate weighted delay across the section are illustrated in Figure 21. Surprisingly, the total delays observed under both early warning systems were lower than those recorded in scenarios without a warning system. Furthermore, this discrepancy widens with an increase in vehicular traffic volume. This could be due to the early warning systems enabling vehicles to slow down progressively ahead of time, thereby decreasing the frequency of stops and alleviating the stop-and-go pattern common in traffic streams. Additionally, this reduces the psychological stress on pedestrians, facilitating more organized and smoother crossing experiences. It is important to highlight that although the VMS warning system allows vehicles to anticipate pedestrian presence and commence deceleration sooner, it lacks the capability for hierarchical warnings. As a result, the concentration of vehicular speed reductions is narrower than with the early warning system, contributing to variations in total delays between the two warning systems. Overall, the total delay associated with the early warning system is less than that of the VMS warning system.

In addition, in the conventional simulation environment in Figure 16, the pedestrian volume was set in gradients ranging from 400 to 1000 ped/h, while the motor vehicle volume was set in gradients ranging from 200 to 500 veh/(h·lane). Three thousand simulations resulted in eight pedestrian–vehicle collisions, with an accident rate of 0.27%. In contrast, in the simulation environment with the pedestrian crossing active safety warning system, 3000 simulations resulted in only two accidents; with an accident rate of 0.06%, the accident rate has significantly decreased. From the above analysis, it can be seen that, in the environment where the pedestrian crossing active safety warning system is used, the vast majority of pedestrians and vehicles will take safe actions such as slowing down or avoiding after observing the intelligent zebra crossing color-change warning. The collision rate between pedestrians and vehicles is greatly reduced. Therefore, the pedestrian crossing active safety warning system has a good two-way warning effect for pedestrians and vehicles, which can improve the safety of pedestrian crossing.

## 6. Conclusions and Prospects

This study addresses the shortcomings of existing stopping sight distance models by proposing a comprehensive safety braking distance model that integrates pedestrian psychological safety with vehicle braking processes. This innovative approach considers pedestrian psychology and allocates varying weights to automobiles and pedestrians, therefore affording pedestrians a certain psychological buffer distance. It alleviates the psychological effects on pedestrians resulting from high vehicle speeds or proximity of parking, ensuring that vehicle braking is more congruent with pedestrian psychological requirements.

An active safety warning system for pedestrian crossings has been developed. This system can track the locations and velocities of pedestrians and cars in real-time, assess potential conflicts between them across multiple scenarios, and execute tailored warning techniques according to specific circumstances. The system achieves bidirectional warnings for both pedestrians and vehicles, enhancing the alertness and reaction speed of traffic participants. Simulation results indicate that after the application of the warning system, the accident rate decreased from 0.27% to 0.06%. This significant reduction demonstrates the system’s effectiveness in improving pedestrian crossing safety through its bidirectional warning mechanism.

Despite the notable achievements in enhancing pedestrian crossing safety, we acknowledge several areas which could be improved:

- The current system primarily relies on millimeter-wave radar and video sensors for detection. Future work could incorporate additional sensor types, such as infrared thermography and LiDAR, to achieve more precise detection of pedestrians and vehicles, thereby increasing the system’s reliability and adaptability.
- The active safety early warning system developed in this research is still in the stage of laboratory simulation and verification, and has not been actually deployed. The construction of simulation environments has inherent limitations, which cannot completely cover or replace all pedestrian crossing scenes in the real world. In the future, we will actively seek the opportunity to cooperate with the urban traffic management department, so as to apply the system to the real environment in the future, and use deep reinforcement learning algorithms to improve the universality of the system and ensure its effective implementation under a wider range of conditions.

## Data Availability

The original contributions presented in this study are included in the article. Further inquiries can be directed to the corresponding author.
